# The Acute Effects of Aerobic Exercise on Nocturnal and Pre-Sleep Arousal in Patients with Unipolar Depression: Preplanned Secondary Analysis of a Randomized Controlled Trial

**DOI:** 10.3390/jcm10174028

**Published:** 2021-09-06

**Authors:** Gavin Brupbacher, Thea Zander-Schellenberg, Doris Straus, Hildburg Porschke, Denis Infanger, Markus Gerber, Roland von Känel, Arno Schmidt-Trucksäss

**Affiliations:** 1 Division of Sports and Exercise Medicine, Department of Sport, Exercise and Health, University of Basel, Birsstrasse 320 B, 4052 Basel, Switzerland; denis.infanger@unibas.ch (D.I.); arno.schmidt-trucksaess@unibas.ch (A.S.-T.); 2 OBERWAID AG, Rorschacher Strasse 311, 9016 St. Gallen, Switzerland; doris.straus@oberwaid.ch (D.S.); hildburg.porschke@oberwaid.ch (H.P.); 3 Department of Psychology, Division of Clinical Psychology and Epidemiology, University of Basel, 4055 Basel, Switzerland; thea.zander@unibas.ch; 4 Division of Sport and Psychosocial Health, Department of Sport, Exercise and Health, University of Basel, Birsstrasse 320 B, 4052 Basel, Switzerland; markus.gerber@unibas.ch; 5 Department of Consultation-Liaison Psychiatry and Psychosomatic Medicine, University Hospital Zurich, Culmannstrasse 8, 8091 Zurich, Switzerland; roland.vonkaenel@usz.ch

**Keywords:** aerobic exercise, depression, heart rate variability, polysomnography, sleep, pre-sleep, arousal

## Abstract

Unipolar depression is associated with insomnia and autonomic arousal. The aim of this study was to quantify the effect of a single bout of aerobic exercise on nocturnal heart rate variability and pre-sleep arousal in patients with depression. This study was designed as a two-arm, parallel-group, randomized, outcome assessor-blinded, controlled, superiority trial. Patients with a primary diagnosis of unipolar depression aged 18–65 years were included. The intervention consisted of a single 30 min moderate-intensity aerobic exercise bout. The control group sat and read for 30 min. The primary outcome of interest was RMSSD during the sleep period assessed with polysomnography. Secondary outcomes were additional heart rate variability outcomes during the sleep and pre-sleep period as well as subjective pre-sleep arousal. A total of 92 patients were randomized to either the exercise (*N* = 46) or the control (*N* = 46) group. Intent-to-treat analysis ANCOVA of follow-up sleep period RMSSD, adjusted for baseline levels and minimization factors, did not detect a significant effect of the allocation (β = 0.12, *p* = 0.94). There was no evidence for significant differences between both groups in any other heart rate variability measure nor in measures of cognitive or somatic pre-sleep arousal. As this is the first trial of its kind in this population, the findings need to be confirmed in further studies. Patients with depression should be encouraged to exercise regularly in order to profit from the known benefits on sleep and depressive symptoms, which are supported by extensive literature.

## 1. Introduction

Insomnia and unipolar depression are bidirectional risk factors for one another [1,2,3,4]. Up to 90% of patients with unipolar depression report symptoms of insomnia [5,6,7,8]. Insomnia has detrimental effects on disease trajectory [9,10,11,12,13]. Insomnia is also the most frequent residual symptom after remission [5,14], which in turn increases the risk of relapse [15,16].

Arousal is a central characteristic and possibly a common pathophysiological mechanism of insomnia and depression. The hyperarousal model postulates that arousal processes in multiple physiological systems cause insomnia [17]. The cognitive model of insomnia hypothesizes that excessive negative thoughts trigger autonomic arousal, which maintains insomnia [18]. The arousal regulation model of affective disorders posits that sensation avoidance and withdrawal in unipolar depression are an autoregulatory reaction to neurophysiologic hyperarousal [19]. Indeed, insomnia [17,20] and depression [21,22,23,24,25,26] have been empirically shown to be associated with somatic, cortical, and cognitive hyperarousal. 

Heart rate variability (HRV) is a universally acknowledged marker for arousal in the context of unipolar depression for multiple reasons. It is well established that a clinical diagnosis of depression and depression severity are associated with lower HRV during the day [27,28,29,30,31] and night [32,33,34,35,36]. Lower HRV is also observed during and immediately after exercise in patients with depression [37]. Moreover, there is evidence to suggest that reduced HRV is an antecedent to depression [38,39]. Reduced HRV is a pathophysiological mechanism [40,41,42,43] explaining the increased risk of cardiovascular disease (CVD) in patients with depression [44,45,46]. 

There is no evidence that psychotherapy and antidepressants, i.e., guideline treatments for depression, increase HRV in this population. On the contrary, tricyclic antidepressants have been unequivocally shown to lower HRV [28,47,48,49,50,51]. Other antidepressants (e.g., selective noradrenaline or serotonin reuptake inhibitors) have produced mixed results, suggesting no effect or a reduction in HRV [28,47,48,49,50,51]. Combined psychotherapy and psychopharmacotherapy do not seem to increase HRV, despite strongly ameliorating depressive symptoms [52]. However, there is evidence that biofeedback [53,54,55] and breathing exercises [56] (as standalone or add-on therapies) increase HRV in patients with depression. Moreover, a considerable portion of patients with depression fail to remit when treated with guideline therapies [57,58,59]. Failure to remit increases the risk for CVD in patients who were initially free of CVD [60,61,62,63,64]. There is a need for further adjuvant therapies that lower arousal during the night in patients with depression, considering the evidence presented above.

Exercise is a promising behavioral adjuvant treatment to lower arousal in depression. Chronic mind–body [65,66] exercise and aerobic exercise [67,68] have been shown to improve HRV in patients with depression. Regular exercise increases HRV in healthy individuals [69,70] and patients with CVD [71,72], as reviews and meta-analyses have shown. We have recently demonstrated that chronic exercise has positive effects on sleep in patients with depression [73]. However, acute bouts of physical activity might have adverse effects. This is reflected in current sleep hygiene recommendations, which state that exercise should not be performed after 2 pm, as this might increase arousal [74]. Findings on the acute effect of moderate-intensity aerobic exercise on the subsequent night’s HRV have been equivocal. While most trials did not detect a difference in nocturnal HRV [75,76,77,78,79,80,81], one study found that moderate aerobic exercise decreased HRV [82]. To our knowledge, there are no trials investigating the acute effects of a single bout of aerobic exercise on HRV in depression. Moreover, we are not aware of any trial investigating the acute or chronic effects of exercise on pre-sleep arousal. Hence, there is a gap in the literature concerning arousal-reducing adjuvant therapies for patients with depression. 

Considering the relevance of sleep for patients with depression outlined above, we chose to study the effects of exercise on nocturnal arousal. Therefore, the primary aim of this trial was to quantify the effect of a single bout of aerobic exercise on nocturnal arousal measured by HRV during sleep in patients with depression. Secondary aims were to investigate intervention effects on pre-sleep HRV and pre-sleep arousal. We hypothesized that the intervention would significantly decrease (1) nocturnal, (2) pre-sleep HRV, and (3) subjective pre-sleep arousal.

## 2. Methods

This study was a two-arm, parallel-group, randomized, controlled, outcome assessor-blinded, superiority trial. We recruited patients within the psychosomatic in-patient rehabilitation unit of the clinic OBERWAID in St. Gallen, Switzerland. The study was conducted in accordance with the Declaration of Helsinki [83], and the Ethics Committee East Switzerland, St. Gallen, Switzerland approved the study protocol (EKOS 18/089). We prospectively registered the trial in the clinicaltrial.gov registry on 17 September 2018 (NCT03673397). We published a detailed study protocol with the study’s rationale [84]. There were no amendments. Analysis of nocturnal HRV deviates from the published protocol. We chose the method presented here because it allows for a more precise analysis than hourly segments after falling asleep. We adhere to the CONsolidated Standards of Reporting Trials (CONSORT) [85] and the Guidelines for Reporting Articles on Psychiatry and Heart rate variability (GRAPH) [86] guidelines in this publication. The data underlying this article are available in the Harvard Dataverse at https://doi.org/10.7910/DVN/WASN36 and will be shared at reasonable request to the corresponding author. The focus of this paper is on the secondary outcomes of HRV, while the primary outcome is presented elsewhere [87]. 

### 2.1. Participants

We screened patients who were admitted to the in-patient psychosomatic rehabilitation unit of the clinic OBERWAID. We conducted the trial in the first five days of the psychosomatic in-patient rehabilitation. The first author or another representative of the clinic OBERWAID obtained written informed consent from all participants involved in the study. Inclusion criteria were: (1) 18–65 years old and (2) a diagnosis of depression (confirmed by experienced psychiatrists according to ICD-10). We applied the following exclusion criteria: (1) regular use of hypnotic agents (patients were included if no hypnotic agents were taken two weeks before study participation), (2) factors precluding exercise testing or training according to the American College of Sports Medicine’s Guidelines for Exercise Testing and Prescription [88], (3) use of beta-blockers (except carvedilol and nebivolol), (4) use of opioids, (5) history of epilepsy, (6) restless legs syndrome (defined by the validated cut-off in the restless legs syndrome screening questionnaire [89]), (7) oxygen desaturation index ≥ 15 (indicative of at least moderate sleep apnea) in the baseline polysomnography, and (8) BMI > 40. We provide a rationale for each criterion in our study protocol [84].

### 2.2. Patient Characteristics

We implemented multiple assessments to characterize patients at baseline. We measured daytime blood pressure as the average of two consecutive measurements on the non-dominant arm while seated after a 5 min resting period. We assessed somatic multimorbidity with a self-assessment and a physician-rated questionnaire. The Patient Health Questionnaire Somatic Symptom Scale (PHQ-15) is a self-administered questionnaire measuring the severity of somatic symptoms (e.g., back pain) within the previous four weeks. It is composed of 15 items on a three-point Likert scale (*0 = not bothered at all to 2 = bothered a lot*). This questionnaire covers 90% of physical complaints reported in outpatient settings. Its validity has been demonstrated [90]. We used the Patient Health Questionnaire-9 (PHQ-9) to assess depressive symptom severity [91]. Nine items (e.g., “feeling down, depressed or hopeless”) are scored on a four-point Likert scale (*0 = not at all to 3 = nearly every day*). The validity of the cut-offs (mild to severe depression) have been demonstrated [91]. We measured anxiety using the Hospital Anxiety and Depression Scale (HADS) [92]. The anxiety subscale contains seven items (e.g., *“I get sudden feelings of panic.”*), each on a four-point Likert scale (e.g., *0 = not at all to 3 = most of the time*). Diagnostic test accuracy and other psychometric properties have been demonstrated [92,93]. We used the Pittsburgh Sleep Quality Index (PSQI) to assess subjective sleep problems [94,95]. Subjective sleep quality, sleep latency, sleep duration, habitual sleep efficiency, sleep disturbances, the use of sleeping medication, and daytime dysfunction are measured with 18 items. The cut-off value of ≥ 5 has been shown to distinguish good from poor sleepers [96]. We assessed sleep reactivity with the Ford Insomnia Response to Stress Test (FIRST) [97,98]. Sleep reactivity is defined as the likelihood of sleep disturbances in response to stressful situations (e.g., *“How likely is it for you to have difficulty sleeping after an argument”*). There are nine items on a four-point Likert scale (*1 = not very likely* to *4 = very likely*). Its reliability and validity have been demonstrated [99]. Lastly, we administered the Dysfunctional Beliefs and Attitudes about Sleep Scale (DBAS) [100]. The sixteen items (e.g., *“I am worried that I may lose control over my ability to sleep.”*) are rated on a Likert scale (*0 = strongly disagree* to *10 = strongly agree*). The reliability and validity of this questionnaire has been demonstrated [100].

We defined the intensity of the intervention based on the individual anaerobic threshold. Consequently, all patients performed a submaximal graded exercise test on a bicycle ergometer (ergoselect 200, Ergoline, Bitz, Germany) before randomization. We determined the anaerobic threshold according to the method of Dickhuth et al. [101] using a specialized software program (Ergonizer, Freiburg, Germany). A detailed description of the graded exercise testing can be found in the study protocol [84].

### 2.3. Randomization

We randomized patients once eligibility was confirmed through baseline polysomnography. We used a nondeterministic unweighted minimization algorithm [102] with a random element of 0.8. The allocation ratio was 1:1. We selected sex, age, depression severity (PHQ-9 score), and subjective sleep quality (PSQI score) as minimization factors. We wanted to ensure the baseline balance of these factors because they are potentially associated with insomnia symptoms or moderate the effects of exercise interventions [103,104,105]. Our allocation concealment consisted of four steps: (1) requesting randomization after baseline measurement, (2) using a random element, (3) requesting randomization by four different study nurses, and (4) not disclosing the full details of minimization to study nurses, in accordance with the Standard Protocol Items Recommendations for Interventional Trials (SPIRIT) guideline [106]. 

### 2.4. Graded Exercise Test, Intervention, and Control

The intervention consisted of a single session of supervised moderate-intensity aerobic exercise on a bicycle ergometer (ergoselect 200, Ergoline, Bitz, Germany). The warm-up period lasted five minutes, with a linear increase from 50% to target intensity. We defined target intensity as 80% of the individual anaerobic threshold (defined by graded exercise testing), i.e., moderate intensity. The duration of the exercise intervention was 30 min. Thereby, the intervention corresponds to the minimum daily physical activity recommendation [107]. We recorded perceived exertion in the 5th, 15th, and 30th minute as well as the average Watt and heart rate (Polar^®^ H7 chest strap, Polar OY, Finland) throughout the intervention. Patients allocated to the control group sat and read magazines when the intervention group was exercising. The exercise and control conditions started at approximately 16:45. 

We implemented several procedures to limit the risk of performance bias. Patients were instructed to refrain from moderate or vigorous exercise on the days of the submaximal exercise test, as well as before and after the polysomnographies. In addition, we assessed contamination through other physical activity with accelerometers. All patients wore a validated [108] (vivofit^®^2, Garmin, Schaffhausen, Switzerland) accelerometer on their non-dominant wrist on the days before and after the polysomnographies. Lastly, the therapy schedule and rules (e.g., timing of meals, consumption of alcohol) of the in-patient rehabilitation clinic limited the variability of many behavioral aspects and ancillary treatments which could influence sleep.

### 2.5. Baseline and Follow-Up Assessments

Outcome assessments at baseline and follow-up were repeated in an identical fashion. Since we could not blind participants in exercise trials, we used objective and subjective measurements to assess arousal.

#### 2.5.1. Polysomnography and Heart Rate Variability

We performed polysomnography with the SOMNOscreen™ plus RC (Somnomedics, Randersacker, Germany) using the following montage: one EEG channel (Fp2-A1, 512 Hz), two EOG channels (1 cm below and 1 cm lateral of the outer right canthus as well as 1 cm above and 1 cm lateral of the outer left outer canthus, 512 Hz), one EMG channel (Chin1–Chin2, 512 Hz), one ECG channel (below the midpoint of the right clavicle and below the left breast crease, in line with the midpoint of the left clavicle, 512 Hz), a thoracic respiratory effort channel (inductance plethysmography belt, 32 Hz), finger photoplethysmography (non-dominant arm, 128 Hz), body position (stored every 30 s), movement (32 Hz), and ambient light (stored every 30 s). Two trained scorers rated sleep stages independently according to the American Association of Sleep Medicine guidelines [109]. Both scorers demonstrated good agreement with the gold standard ratings in the AASM inter-scorer program [110]. Scorers were blinded against allocation, time point, and each other’s ratings. Participants were instructed to lay in bed for at least five more minutes after they woke up.

ECG pre-processing and HRV analysis was carried out while being blinded against allocation and time point using Kubios HRV (Version 3.4.2) (University of Eastern Finland, Kuopio, Finland) [111]. The validity of Kubios HRV has been demonstrated [112]. QRS detection was based on the Pan-Tompkins algorithm [113], including bandpass filtering. Artifacts were identified using a validated algorithm [114]. We visually inspected beat detection, manually adding missed beats when necessary. Ectopic beats were replaced by phantom beats using cubic spline interpolated RR values. Detrending was performed using the smoothness priors approach (λ  =  500, fc =  0.035 Hz) [115]. We computed power spectral density using Lomb–Scargle periodogram (LSP) [116,117] with a moving average filter (width 0.02 Hz). We chose LSP instead of Fast Fourier Transformation (FFT) and autoregressive modeling (AR) for multiple reasons. FFT and AR require resampling (thereby introducing bias [118,119]) and a trade-off between frequency resolution and time resolution [120,121]. LSP, however, makes no assumptions of models, is more accurate [122,123,124], is less noisy [125], has higher reliability [126], and is more sensitive to physiological changes [125,126,127] compared to FFT. Based on the aforementioned specifications, we report low-frequency power (LF, 0.04–0.15 Hz [ms2]), high-frequency power (HF, 0.15–0.4 Hz [ms2]), and the LF/HF ratio [128]. Time-domain parameters include the heart rate, the root mean square of successive differences of normal-to-normal intervals (RMSSD), and the standard deviation of all normal-to-normal intervals (SDNN). Although there is an ongoing debate about the physiological correlates of some HRV variables, RMSSD and HF are generally accepted to be measures of vagal modulation [129,130].

We assessed HRV (1) during the last 5 min segment before the first epoch of any sleep stage, (2) during the sleep period (i.e., from the first to the last episode of any sleep stage), and (3) during each sleep stage. The methodological details and the rationale for this choice are presented in Table S1. We excluded any 5 min segment from the analysis which had either ≥5% artifacts or in which the patient was upright. Correction for heart rate was not necessary, since the heart rate did not differ between both groups in any of the HRV analyses. Sleep period RMSSD was the primary HRV marker of interest, since it has a clear physiological interpretation in the context of arousal (i.e., vagal tone).

#### 2.5.2. Pre-Sleep Arousal

We asked participants to complete the Pre-Sleep Arousal Scale [131] upon awakening from baseline and follow-up nights. The Pre-Sleep Arousal Scale assesses cognitive (eight items) and somatic (seven items) pre-sleep arousal symptoms. Patients rate how intensely they experienced each of the symptoms as they attempted to fall asleep. All 15 items (e.g., “a jittery, nervous feeling in your body”) are scored on a five-point Likert scale (*1* = *not at all* to *5* = *extremely*) and summed up for each factor separately [131,132]. 

### 2.6. Statistical Methods

We examined whether there was a first-night effect in the control group. Since we found no first-night effect, we included baseline and follow-up measures in the analysis using an ANCOVA model [133]. We used baseline outcome and minimization factors [134] as covariates, allocation as the independent variable, and follow-up outcome as the dependent variable. We checked all statistical prerequisites. We computed robust standard errors in the case of heteroscedastic residuals (using HC3) [135]. Predefined sensitivity analyses for the primary outcome of interest (i.e., follow-up RMSSD) were performed to gauge the influence of several factors: influential data points, smoking status, as well as use of beta-blockers, any class of antidepressants, and tricyclic antidepressants. We used multiple imputation with predictive mean matching to replace missing values [136] and conducted intent-to-treat analyses. The missing completely at random assumption was met using Little’s test [137]. All analyses were performed using the software R, version 3.6.3 [138].

Sample size calculation was based on another outcome (i.e., sleep efficiency, see study protocol [84] for comprehensive details). We did not perform an a priori or post hoc sample size calculation for secondary outcomes.

## 3. Results

**Figure 1:**
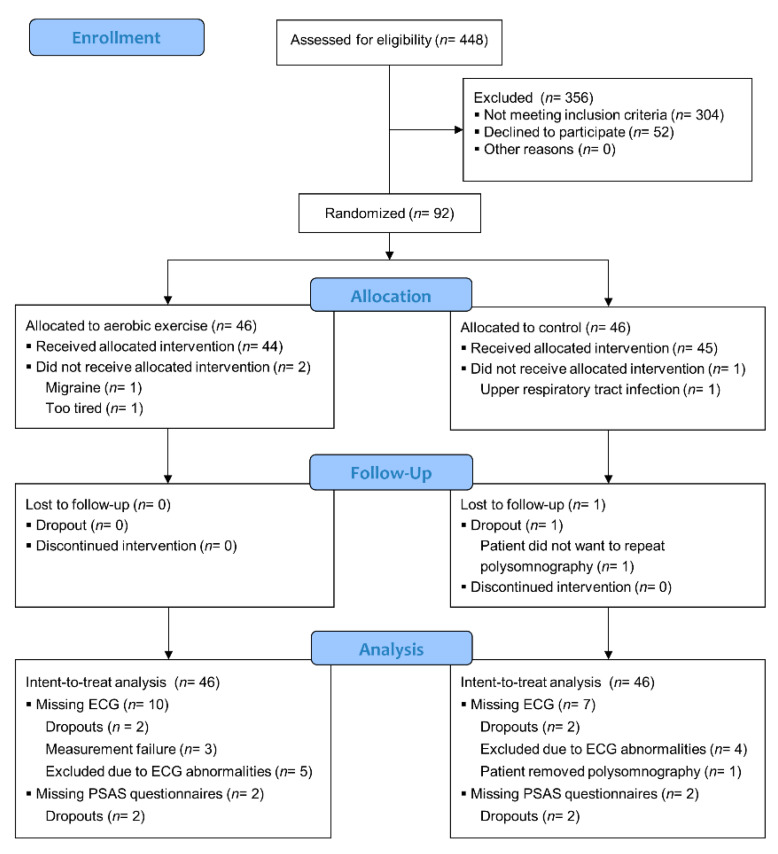
CONSORT participant flow.

We screened 448 patients between September 2018 and January 2020. We randomized 92 patients to either the aerobic exercise intervention (*N* = 46) or the control condition (*N* = 46), as shown in Figure 1. Reasons for data loss included dropouts (*N* = 2 in each group), ECG measurement failure (*N* = 3 in the intervention arm), and a patient who removed the polysomnographic equipment during the night (*N* = 1 in the control group). We excluded multiple patients due to ECG abnormalities (*N* = 5 and *N* = 4 in intervention and control arm, respectively). This resulted in *N* = 36 and *N* = 39 complete ECG datasets (i.e., baseline and follow up) for the intervention and control arm, respectively. During the sleep period (sleep onset until the last awakening), the average amount of corrected beats was 0.34% (range: 0.02–2.13%). Baseline characteristics of the study sample are presented in Table 1. As reported previously for this study [87], the inter-rater reliability of polysomnographic scoring was good, and the daily steps during the trial as well as the rate and intensity of adverse events did not differ between the groups.

### 3.1. Sleep Period

**Figure 2:**
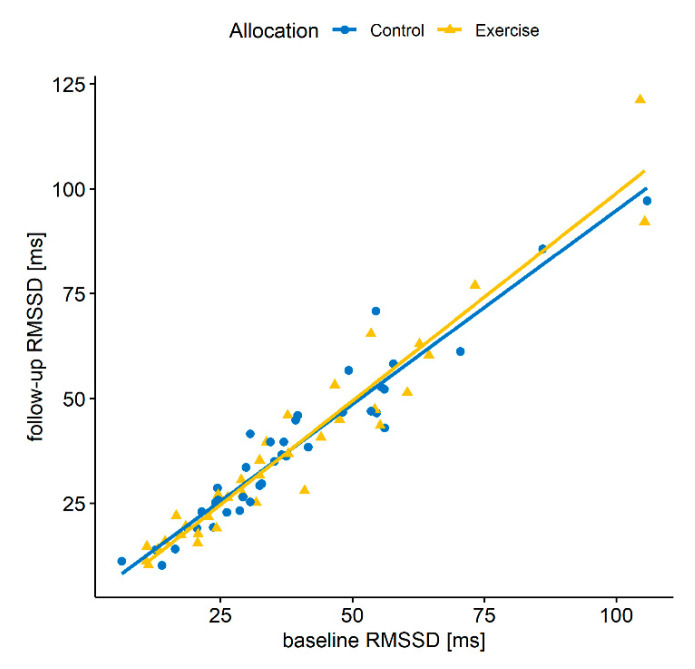
Baseline and follow-up RMSSD by allocation.

There was no evidence that heart rate differed between the groups during the sleep period (β  =  −0.43, 95% CI: −1.44–0.58, *p* = 0.40). The intent-to-treat analysis ANCOVA of follow-up RMSSD, adjusted for pre-intervention levels and minimization factors, did not detect a significant effect of the allocation during the sleep period (see Table 2 and Figure 2). The difference between the mean change scores (baseline to follow up) of both groups corresponds to the coefficient for allocation (see Table 2). This finding was confirmed in all pre-specified sensitivity analyses (i.e., only using complete data, excluding patients who smoked, used either beta-blockers, any antidepressant, or only tricyclic antidepressants). We identified one influential data point using Cook’s distance and DFBETAs, but excluding this data point did also not alter the finding.

There was no evidence that any of the other HRV outcomes were affected through the intervention during the sleep period (see Table 3). Sensitivity analyses based on complete data only, excluding patients who smoked, used either beta-blockers, any antidepressant, or only tricyclic antidepressants, also confirmed these results. 

### 3.2. Sleep Stages

We found no evidence that heart rate differed during any sleep stage (N2: β  =  −0.70, 95% CI: −2.26–0.85, *p* = 0.37; N3: β  =  −0.21, 95% CI: −2.64–2.22, *p* = 0.86; non-REM: β  =  −0.40, 95% CI: −1.71–0.91, *p* = 0.54; REM: β  =  −0.40, 95% CI: −2.47–1.67, *p* = 0.70). There was no evidence that the aerobic exercise intervention affected RMSSD during stage two or stage three sleep, nor during REM or non-REM sleep (see Table 4). Since most patients did not have an uninterrupted 10 min segment of N1 sleep on both nights needed for this analysis, we could not perform the analysis for this stage of sleep. 

### 3.3. Pre-Sleep

We had to exclude seven measurements (*N* = 4 at baseline, *N* = 3 at follow-up) in addition to the missing data reported in Figure 1 from this analysis because these segments either had ≥5% artifacts, the patient was upright, or the patient fell asleep within the segment. We did not find any evidence that the intervention altered pre-sleep heart rate (β  =  0.53, 95% CI: −3.03–4.08, *p* = 0.77) or pre-sleep HRV (see Table 5).

Cronbach’s α of the cognitive (baseline  =  0.87; follow up = 0.90) and somatic (baseline  =  0.71; follow up = 0.78) pre-sleep arousal subscales were good. Intent-to-treat ANCOVAs of follow-up pre-sleep arousal, adjusted for baseline levels and minimization factors, also provided no evidence that the intervention altered somatic (β  =  −0.65, 95% CI: −2.04–0.74, *p* = 0.35) or cognitive (β  =  −0.15, 95% CI: −2.08–1.79, *p* = 0.88) pre-sleep arousal.

## 4. Discussion

The primary goal of this trial was to quantify the effect of a single bout of 30 min moderate aerobic exercise on arousal measured by HRV during sleep in patients with depression. We did not find evidence that the intervention affected HRV during the sleep period, nor in specific sleep stages. The absence of evidence was very robust. The findings were confirmed across all outcome measures of HRV and in all of the sensitivity analyses.

To the best of our knowledge, this is the first trial to investigate the effects of a single bout of aerobic exercise on arousal measured by HRV during sleep in patients with depression. Trials in primarily young and healthy individuals have found mixed results. There are diverging findings concerning the duration of an exercise intervention on HRV in the subsequent night. Myllymäki et al. [139] found that an incremental bicycle ergometer exercise until exhaustion lasting approximately 30 min did not alter HRV. This was confirmed by another study, which showed that moderate-intensity aerobic exercise sessions lasting 30 or 60 min had no effect, while sessions of 90 min decreased nocturnal HRV [75]. Trials concerning the effect of exercise intensity have also produced inconsistent results. Except for one study [82], multiple [75,76,77,78,79,80,81] trials found no evidence that moderate-intensity exercise altered HRV during the night. Five trials that directly compared moderate and vigorous exercise intensities on nocturnal HRV found no difference [75,76,77,78,79], whereas two trials found that high-intensity exercise reduced nocturnal HRV [80,81]. Another two trials only comparing high-intensity exercise with a control condition also found that the intervention reduced nocturnal HRV [79,140]. The effects of exercise timing during the day are equally inconclusive. In healthy young males, exercising vigorously on three consecutive days in the morning altered HRV (increasing LF and HF) outcomes during sleep, but exercising in the evening did not have a discernable effect [141]. However, Ramos-Campo et al. [77] found no evidence that morning or evening exercise affected HRV during sleep in trained individuals. However, prolonged and intense exercise such as a marathon [82] or a 75 km cross-country skiing race [142] have been shown to reduce HRV during the following night. Many of these trials investigating the acute effects of exercise on sleep were conducted with trained or healthy individuals. HRV in athletes tends to be higher [143], whereas in patients with unipolar depression, HRV tends to be lower [30] compared with healthy controls. This should be taken into account when interpreting these results. Acute effects of aerobic exercise on HRV during sleep might depend on exercise variables such as intensity, duration, volume, and timing during the day, as well as the training status of study participants. Our results are in line with the findings presented above, i.e., there is no evidence that a single moderate aerobic exercise session of 30 min performed multiple hours before bedtime is a strong enough stimulus to alter HRV in the subsequent night. All of these trials analyzed nocturnal HRV in fixed periods without accounting for the different sleep phases. 

We are aware of only one other trial which analyzed the acute effects of aerobic exercise on HRV during different sleep phases. Yamanaka et al. [141] subjected healthy young males to a 7-day dim light (<10 lux) protocol. Subjects performed vigorous-intensity aerobic exercise during the morning or afternoon on three consecutive days for 90 min each day (10 min of warming up, 45 min of exercise, 10 min of rest on a chair, 45 min of exercise, and 10 min of cooling down). After three days, the morning group had higher values in LF and HF during N1 and N2 as well as higher values in HF during N3. We did not find any evidence that the intervention altered HRV during sleep stages N2 and N3, as well as non-REM and REM sleep. However, sleep stage N1 could not be analyzed. Possible reasons for theses diverging findings might be the dim light setting, the repeated exercise stimulus over three days, and the specification of exercise variables (90 min at vigorous intensity).

We did not find evidence that a single bout of moderate-intensity aerobic exercise altered objectively or subjectively measured pre-sleep arousal. We did not detect an effect of the intervention on any of the HRV indices measured in the last 5 min before the first epoch of sleep. Furthermore, there is no evidence to suggest that the somatic or cognitive pre-sleep arousal questionnaire subscales differed between the groups. This is the first trial to investigate the effect of aerobic exercise on pre-sleep arousal in patients with depression or any other group of participants, to the best of our knowledge. 

Oda and Shirakawa [76] investigated the acute effects of aerobic exercise on ‘emotional comfort’ before falling asleep—an outcome potentially similar to pre-sleep arousal. An amount of 40 min of moderate or vigorous exercise (ending one hour before going to bed) led to higher ‘emotional comfort’ compared to the control condition. Although the construct of ‘emotional comfort’ might be related to pre-sleep arousal, these findings are not transferable to clinical populations because the trial only included healthy participants. Theoretical considerations and data from previous trials suggest that reducing pre-sleep arousal, as measured by HRV, might be beneficial in people with insomnia symptoms. Guidelines recommend cognitive behavioral therapy for insomnia as a first-line therapy [144], to, amongst other effects, reduce pre-sleep arousal [145]. There is some evidence that biofeedback interventions that reduce pre-sleep arousal positively affect cardiac autonomic control during the night [146,147] and objectively measured sleep quality [147,148].

These findings have implications for clinical practice and research. While our results do not suggest that a single session of moderate-intensity aerobic exercise ameliorates nocturnal HRV, they are in line with previous studies which failed to detect a negative effect on nocturnal HRV. This contradicts the current sleep hygiene recommendations [74]. These recommendations state that rigorous exercise might release endorphins, thereby hindering sleep onset [74]. The evidence from our study suggests that moderate-intensity aerobic exercise after 2 pm can be cautiously recommended as far as autonomic, cognitive, and somatic arousal are concerned. Moreover, previous trials in healthy individuals found no adverse effects on sleep from an acute exercise bout in the evening [149]. Considering that arousal might be a common pathophysiological mechanism in insomnia and depression and the paucity of literature concerning interventions that can reduce arousal, future studies are needed. Such trials should try to identify interventions that can reduce arousal, e.g., biofeedback or mindfulness-based stress reduction. Trials specifically investigating exercise should focus on the effect that intensity, duration, and timing have on HRV or other measures of arousal. More broadly, HRV has been recognized as an index of self-regulation [150] and as a transdiagnostic biomarker of psychopathology [151]. Hence, in future studies, it would be interesting to study the effects of exercise on HRV in the context of self-regulation and interoception [152].

This study has several strengths and limitations. The risk of bias was limited through multiple procedures: allocating patients using minimization with appropriate allocation concealment (selection bias), blinding outcome assessors during polysomnography rating and HRV data analysis (detection bias), avoiding contamination through other physical activity and extraneous factors (performance bias), and using intent-to-treat analysis (attrition bias). However, the external validity of this trial might be limited by two factors: the exclusion of patients who used hypnotics (although this increases internal validity) and the relatively normal levels of polysomnographically measured sleep variables (see Table 1). Both these factors may not be representative of typical patients with depression. The a priori sample size calculation for this study was not calculated for sleep period RMSSD, but for another outcome which we have reported elsewhere [87]. Lastly, the menstrual cycle was not considered as a potentially confounding variable. However, it seems unlikely that this factor has influenced the results since the duration of the trial was very short (i.e., measurements were conducted on two consecutive nights) and the statistical analyses adjusted for baseline values. However, we cannot definitively exclude the possibility that the menstrual cycle may have influenced susceptibility to exercise-induced HRV changes.

## 5. Conclusions

We found no evidence that a single 30 min bout of moderate-intensity aerobic exercise affected pre-sleep or nocturnal arousal reflected by indices of HRV. Our findings need to be interpreted cautiously, considering that this is the first trial of this nature in patients with depression. The evidence base remains insufficient to explicitly recommend exercising in the late afternoon or evening hours to ameliorate sleep. Non-inferiority trials and studies investigating the interplay of exercise intensity, duration, and timing, with patient characteristics (e.g., chronotype) are needed. This would further our understanding, allowing for the formulation of personalized exercise prescriptions. 

## Figures and Tables

**Table 1:** Baseline characteristics.

		Intervention Group (*N* = 46)	Control Group (*N* = 46)
Age		46 (37, 53)	48 (43, 51)
Sex	female	32 (70)	33 (72)
	male	14 (30)	13 (28)
BMI		25.0 (21.8, 29.2)	24.4 (22.2, 27.7)
Systolic blood pressure (mmHg)		121 (115, 131)	121 (112, 134)
Diastolic blood pressure (mmHg)		80 (74, 88)	81 (72, 87)
Smoking	never smoked	26 (57)	24 (52)
	stopped smoking since ≥12 months	8 (17)	10 (22)
	currently smoking or stopped since <12 months	12 (26)	12 (26)
Alcohol	less than 1 day/week	5 (15)	6 (18)
	1–2 days/week	14 (41)	6 (18)
	3–6 days/week	7 (21)	12 (37)
	daily	8 (23)	9 (27)
PHQ15		12.5 (8.3, 15.8)	12.5 (8.0, 14.0)
PHQ9		14.0 (12.0, 17.0)	15.0 (12.0, 17.0)
HADS anxiety		11.0 (9.3, 14.0)	11.5 (9.0, 14.0)
Antidepressant medication		19 (41)	21 (50)
DBAS		4.6 (3.6, 5.7)	4.8 (3.8, 5.4)
FIRST		27.0 (24.0, 29.8)	27.5 (21.3, 29.8)
PSQI		10.0 (7.00, 13.8)	9.5 (6.3, 12.0)
Sleep efficiency ^a^		91.3 (84.4, 93.5)	88.8 (82.5, 94.0)
Total sleep time ^a^		439.0 (393.3, 479.5)	416.7 (382.3, 463.1)
Sleep onset latency ^a^		14.0 (5.5, 23.3)	14.5 (6.8, 27.2)
Wake after sleep onset ^a^		30.8 (18.0, 43.3)	37.8 (19.1, 62.6)
Number of awakenings ^a^		17.0 (13.5, 23.5)	15.8 (11.4, 20.8)
RMSSD		32.1 (20.7, 49.0)	34.4 (24.5, 51.4)
SDNN		37.8 (27.2, 55.0)	34.7 (29.5, 52.6)
LF (ms^2^)		858 (474, 1659)	780 (436, 1852)
HF (ms^2^)		382 (159, 925)	410 (253, 810)
Somatic pre-sleep arousal		10.0 (9.0, 12.8)	12.0 (9.0, 15.0)
Cognitive pre-sleep arousal		15.0 (13.3, 18.0)	15.0 (11.0, 20.0)

Note: Continuous variables are presented as medians with interquartile ranges (median (Q_1_, Q_2_)) and categorical variables are presented as absolute numbers and percentages (N (%)). ^a^ measured polysomnographically. Abbreviations: BMI = body mass index; PHQ15 = Patient Health Questionnaire 15; PHQ9 = Patient Health Questionnaire 9; HADS anxiety: Hospital Anxiety and Depression, anxiety subscale; DBAS = Dysfunctional Beliefs and Attitudes about Sleep Scale; FIRST = Ford Insomnia Response to Stress Test; PSQI = Pittsburgh Sleep Quality Index; RMSSD = root mean square of successive differences between normal heartbeats; SDNN = standard deviation of normal-to-normal RR intervals; LF = low-frequency power (0.04–0.15 Hz (ms^2^)); HF = high-frequency power (0.15–0.4 Hz (ms^2^)).

**Table 2:** ANCOVA table for intent-to-treat analysis of RMSSD during sleep period at follow-up.

Term	Estimate (β)	Standard Error (β)	95% Confidence Interval		*p*-Value
Intercept	6.78	6.03	−5.42	18.98	0.27
Baseline RMSSD	0.93	0.04	0.84	0.84	0.001
Age	−0.10	0.10	−0.30	0.10	0.61
Sex (male ^a^)	1.21	1.70	−2.22	4.64	0.48
PHQ9	0.08	0.17	−0.26	0.42	0.64
PSQI	−0.16	0.18	−0.51	0.19	0.37
Allocation (exercise ^b^)	0.12	1.53	−2.98	3.22	0.94

Note: age, sex, PHQ9, and PSQI were entered as covariates because they were used as minimization factors. Abbreviations: RMSSD = root mean square of successive differences between normal heartbeats; PHQ9: Patient Health Questionnaire 9; PSQI: Pittsburgh Sleep Quality Index. ^a^ Sex was coded as follows: 1 = male, 2 = female. ^b^ Exercise was coded as follows: 1 = control, 2 = exercise.

**Table 3:** Coefficients of exercise allocation in intent-to-treat analysis ANCOVA models predicting HRV outcomes during sleep period.

Outcome	$\mathrm{Estimate}\;\mathrm{for}\;\mathrm{Exercise}\;\mathrm{Allocation}{\;}^{a}\;(\beta )$	Standard Error (β)	95% Confidence Interval		*p*-Value
SDNN	−0.27	1.78	−4.08	3.53	0.88
LF	−130	260	−791	531	0.64
HF	16	75	−135	167	0.83
LF/HF-ratio	−0.17	0.34	−0.85	0.51	0.62

Note: All models used baseline values of the outcome as well as minimization factors (sex, age, PHQ9 score, and PSQI score) as covariates and allocation as the independent variable. The coefficient for allocation is the difference of the mean change score in the exercise group compared to the control group. Abbreviations: SDNN = standard deviation of normal-to-normal RR intervals; LF = low-frequency power (0.04–0.15 Hz (ms^2^)); HF = high-frequency power (0.15–0.4 Hz (ms^2^)). ^a^ Allocation was coded as follows: 1 = control, 2 = exercise.

**Table 4:** Coefficients of exercise allocation in intent-to-treat ANCOVA models predicting RMSSD outcomes during different sleep stages.

Sleep Stage	$\mathrm{Estimate}\;\mathrm{for}\;\mathrm{Exercise}\;\mathrm{Allocation}{\;}^{a}\;(\beta )$	Standard Error (β)	95% Confidence Interval		*p*-Value
N2	−0.69	4.75	−10.41	9.02	0.88
N3	−7.15	7.11	−23.10	8.81	0.34
non-REM	0.29	1.81	−3.32	3.90	0.87
REM	−4.93	8.04	−22.42	12.55	0.55

Note: All models used baseline values of the outcome as well as minimization factors (sex, age, PHQ9 score, and PSQI score) as covariates and allocation as the independent variable. The coefficient for allocation is the difference of the mean change score in the exercise group compared to the control group. Abbreviations: N2 = stage two sleep, N3 = stage three sleep, REM = rapid eye movement sleep. ^a^ Allocation was coded as follows: 1 = control, 2 = exercise.

**Table 5:** Coefficients of exercise allocation in intent-to-treat ANCOVA models predicting HRV outcomes during the pre-sleep period.

Outcome	$\mathrm{Estimate}\;\mathrm{for}\;\mathrm{Exercise}\;\mathrm{Allocation}{\;}^{a}\;(\beta )$	Standard Error (β)	95% Confidence Interval		*p*-Value
RMSSD	0.21	2.61	−4.99	5.41	0.94
SDNN	−1.00	2.58	−6.15	4.15	0.70
LF	34	155	−274	343	0.83
HF	100	130	−158	358	0.44
LF/HF-ratio	1.33	1.05	−0.76	3.43	0.21

Note: All models used baseline values of the outcome as well as minimization factors (sex, age, PHQ9 score, and PSQI score) as covariates and allocation as the independent variable. The coefficient for allocation is the difference of the mean change score in the exercise group compared to the control group. Abbreviations: RMSSD = root mean square of successive differences between normal heartbeats; SDNN = standard deviation of normal-to-normal RR intervals; LF = low-frequency power (0.04–0.15 Hz (ms^2^)); HF = high-frequency power (0.15–0.4 Hz (ms^2^)). ^a^ Allocation was coded as follows: 1 = control, 2 = exercise.

## Data Availability

The data underlying this article are available in the Harvard Dataverse at https://doi.org/10.7910/DVN/WASN36 and will be shared on reasonable request to the corresponding author.

## Supplementary Materials

The following are available online at https://www.mdpi.com/article/10.3390/jcm10174028/s1, Table S1: Time spans for which HRV was calculated and the rationale.
