# Supplementary material for: The Acute Effects of Aerobic Exercise on Nocturnal and Pre-Sleep Arousal in Patients with Unipolar Depression: Preplanned Secondary Analysis of a Randomized Controlled Trial
Source: J Clin Med. 2021 Sep 6;10(17):4028. doi: 10.3390/jcm10174028 (PMC8432550; doi:10.3390/jcm10174028)
Supplement: Supplementary file 1 [file jcm-10-04028-s001.zip › jcm-1271829-supplementary.pdf]

Supplementary

Table S1. Time spans for which HRV was calculated and the rationale.

Time span	Operationalization	Rationale
Pre-sleep	The 5-minute segment before the first epoch of any sleep stage	Theoretical considerations [1,2] and empirical data suggest that reducing pre-sleep arousal can ameliorate sleep [3–5].
Sleep period	The average of consecutive non-overlapping 5-min segments starting from the beginning of the first epoch of sleep to the end of the last epoch of sleep (i.e., including periods of wakefulness)	Depression is associated with lower HRV during the night [6–10].
Sleep stages N1, N2, N3, and REM	Only 5-min segments consisting of consecutive epochs of a particular sleep stage (N1, N2, N3, or REM) were included in the analysis. The last consecutive 5-minute segment of a particular sleep stage was excluded since changes in HRV have been shown to precede sleep stage changes [11–14]. Hence, only phases containing at least 10 minutes with consecutive epochs of a particular sleep stage were used to extract 5-min segments for the analysis. We computed the average of all 5-min segments for each sleep stage. This is analogous to the method used by Herzig et al. 2018 [15].	We differentiate individual sleep stages as well as non-REM and REM sleep since these sleep stages also differ in terms of parasympathetic and sympathetic predominance [14].

References

1. Harvey, A.G. A Cognitive Model of Insomnia. *Behav Res Ther* **2002**, *40*, 869–893.
2. Ong, J.C.; Ulmer, C.S.; Manber, R. Improving Sleep with Mindfulness and Acceptance: A Metacognitive Model of Insomnia. *Behav Res Ther* **2012**, *50*, 651–660, doi:10.1016/j.brat.2012.08.001.
3. Sakakibara, M.; Hayano, J.; Oikawa, L.O.; Katsamanis, M.; Lehrer, P. Heart Rate Variability Biofeedback Improves Cardiorespiratory Resting Function during Sleep. *Appl Psychophysiol Biofeedback* **2013**, *38*, 265–271, doi:10.1007/s10484-013-9232-7.
4. de Zambotti, M.; Sizintsev, M.; Claudatos, S.; Barresi, G.; Colrain, I.M.; Baker, F.C. Reducing Bedtime Physiological Arousal Levels Using Immersive Audio-Visual Respiratory Bio-Feedback: A Pilot Study in Women with Insomnia Symptoms. *J Behav Med* **2019**, *42*, 973–983, doi:10.1007/s10865-019-00020-9.
5. Ebben, M.R.; Kurbatov, V.; Pollak, C.P. Moderating Laboratory Adaptation with the Use of a Heart-Rate Variability Biofeedback Device (StressEraser). *Appl Psychophysiol Biofeedback* **2009**, *34*, 245–249, doi:10.1007/s10484-009-9086-1.
6. Yang, A.C.; Tsai, S.-J.; Yang, C.-H.; Kuo, C.-H.; Chen, T.-J.; Hong, C.-J. Reduced Physiologic Complexity Is Associated with Poor Sleep in Patients with Major Depression and Primary Insomnia. *Journal of Affective Disorders* **2011**, *131*, 179–185, doi:10.1016/j.jad.2010.11.030.
7. Leistedt, S.J.-J.; Linkowski, P.; Lanquart, J.-P.; Mietus, J.E.; Davis, R.B.; Goldberger, A.L.; Costa, M.D. Decreased Neuroautonomic Complexity in Men during an Acute Major Depressive Episode: Analysis of Heart Rate Dynamics. *Translational Psychiatry* **2011**, *1*, e27–e27, doi:10.1038/tp.2011.23.
8. Saad, M.; Ray, L.B.; Bujaki, B.; Parvaresh, A.; Palamarchuk, I.; De Koninck, J.; Douglass, A.; Lee, E.K.; Soucy, L.J.; Fogel, S.; et al. Using Heart Rate Profiles during Sleep as a Biomarker of Depression. *BMC Psychiatry* **2019**, *19*, 168, doi:10.1186/s12888-019-2152-1.
9. Pawlowski, M.A.; Gazea, M.; Wollweber, B.; Dresler, M.; Holsboer, F.; Keck, M.E.; Steiger, A.; Adamczyk, M.; Mikoteit, T. Heart Rate Variability and Cordance in Rapid Eye Movement Sleep as Biomarkers of Depression and Treatment Response. *J Psychiatr Res* **2017**, *92*, 64–73, doi:10.1016/j.jpsychires.2017.03.026.
10. Kwon, H.B.; Yoon, H.; Choi, S.H.; Choi, J.-W.; Lee, Y.J.; Park, K.S. Heart Rate Variability Changes in Major Depressive Disorder during Sleep: Fractal Index Correlates with BDI Score during REM Sleep. *Psychiatry Research* **2019**, *271*, 291–298, doi:10.1016/j.psychres.2018.11.021.
11. Jurysta, F.; van de Borne, P.; Migeotte, P.-F.; Dumont, M.; Lanquart, J.-P.; Degaute, J.-P.; Linkowski, P. A Study of the Dynamic Interactions between Sleep EEG and Heart Rate Variability in Healthy Young Men. *Clin Neurophysiol* **2003**, *114*, 2146–2155, doi:10.1016/s1388-2457(03)00215-3.
12. Long, X.; Arends, J.B.; Aarts, R.M.; Haakma, R.; Fonseca, P.; Rolink, J. Time Delay between Cardiac and Brain Activity during Sleep Transitions. *Appl. Phys. Lett.* **2015**, *106*, 143702, doi:10.1063/1.4917221.
13. Jurysta, F.; Kempenaers, C.; Lancini, J.; Lanquart, J.-P.; van de Borne, P.; Linkowski, P. Altered Interaction between Cardiac Vagal Influence and Delta Sleep EEG Suggests an Altered Neuroplasticity in Patients Suffering from Major Depressive Disorder. *Acta Psychiatr Scand* **2010**, *121*, 236–239, doi:10.1111/j.1600-0447.2009.01475.x.

14. de Zambotti, M.; Trinder, J.; Silvani, A.; Colrain, I.M.; Baker, F.C. Dynamic Coupling between the Central and Autonomic Nervous Systems during Sleep: A Review. *Neuroscience & Biobehavioral Reviews* **2018**, *90*, 84–103, doi:10.1016/j.neubiorev.2018.03.027.
15. Herzig, D.; Eser, P.; Omlin, X.; Riener, R.; Wilhelm, M.; Achermann, P. Reproducibility of Heart Rate Variability Is Parameter and Sleep Stage Dependent. *Front Physiol* **2018**, *8*, doi:10.3389/fphys.2017.01100.
